# The complete mitochondrial genome of *Pristurus rupestris rupestris*

**DOI:** 10.1080/23802359.2017.1398612

**Published:** 2017-11-09

**Authors:** Pedro Tarroso, Marc Simó-Riudalbas, Salvador Carranza

**Affiliations:** a CIBIO/InBio, Centro de Investigação em Biodiversidade e Recursos Genéticos, Laboratório Associado, Universidade do Porto, Vairão, Portugal;; b Institute of Evolutionary Biology (CSIC-Universitat Pompeu Fabra), Barcelona, Spain

**Keywords:** *Pristurus rupestris*, Sphaerodactylidae, mitogenome

## Abstract

*Pristurus rupestris rupestris* is a gecko of the family Sphaerodactylidae adapted to the arid habitat found in the Hajar Mountains of southeastern Arabia. The complete mitochondrial genome was obtained with Illumina sequencing. The sequenced mitogenome has 13 protein-coding genes, 22 tRNAs, two rRNA genes and two non-coding regions, totalling 16,993 bp. The AT content of the obtained sequence is 52.1% (A:28.7%, T:23.4%, G:14.7%, C:33.2%). The control region has an AT content of 54.3% and a length of 1558 bp.

*Pristurus rupestris rupestris* is a species complex with genetically divergent but morphologically cryptic populations living in allopatry along its range in the Hajar Mountains of southeastern Arabia (Badiane et al. 2014; Garcia-Porta et al. 2017). It belongs to the family Sphaerodactylidae, which is the second most diverse family within Gekkota. The recent sequencing techniques provide an affordable and simple path to fully sequence the mitochondrial genome and multiple gecko’s mitogenomes have been published. Yet, the Sphaerodactylidae family is poorly represented with only three mitogenomes belonging to two different species of the genus *Teratoscincus* available from GenBank (search with keyword Gekkota, June 2017). Here, we provide the first mitogenome from the genus *Pristurus* available in a public sequence database.

The individual was collected on 8 October 2010 (Muscat; 23.616°N, 58.585°E) at the type locality of the species (Badiane et al. 2014). The specimen was preserved in ethanol in the collection of the Institute of Evolutionary Biology (reference IBES7709). Total DNA was extracted using the DNeasy Blood & Tissue Kit (Qiagen, Valencia, CA). The library was prepared using the TruSeq DNA PCR-Free library and sequenced in a HiSeqX Ten sequencer for 2 × 150 bp (350 bp insert size), resulting in over 770 million reads. The adapters were removed with Trimmomatic v0.36 (Bolger et al. 2014), preserving paired sequences with quality above 15 (4 bp sliding window). The dataset was randomly sampled to 20 parts with a custom python script (Supplementary material) from which mitogenomes were extracted with MITObim v1.9 (Hahn et al. 2013) using a 12S sequence seed from the same individual (accession no. KY023600). Using a python script (Supplementary material) we extracted the circular part of each of the 20 sequences. We used MARS (Ayad and Pissis 2017) to rotate the sequences to the same origin, aligned with MAFFT v7.307 (Katoh and Standley 2013) and got the consensus with EMBOSS suite v6.6.0 ‘cons’ tool. Lack of consensus resulted in ambiguous bases that were removed in the final sequence. We used MITOS webserver rev.917 (Bernt et al. 2013) for preliminary annotation and manually checked in Unipro UGENE v1.26.3 (Okonechnikov et al. 2012).

The consensus sequence for the mitochondrial genome (GenBank accession no. MG182397) is 16,993 bp long. The AT content of the obtained sequence is 52.1% with the base composition as A:28.7%, T:23.4%, G:14.7%, and C:33.2%. There are 13 protein-coding genes, 22 tRNA, two rRNA and two non-coding regions, following standard vertebrate arrangement pattern. The two non-coding regions, the control region and replication origin, are 1558 and 27 bp long, respectively. We inferred a phylogenetic tree comprising representative genera of Gekkota families with available mitogenomes in GenBank (Figure 1). Phylogenetic analyses were performed under Maximum Likelihood and Bayesian Inference frameworks (see Supplementary material). The phylogenetic inference supports previously known relationships between families, except monophyly of Gekkonidae. The newly sequenced *Pristurus r. rupestris* appears sister taxon to the available sphaerodactilid *Teratoscincus keyserlingii*.

**Figure 1.**
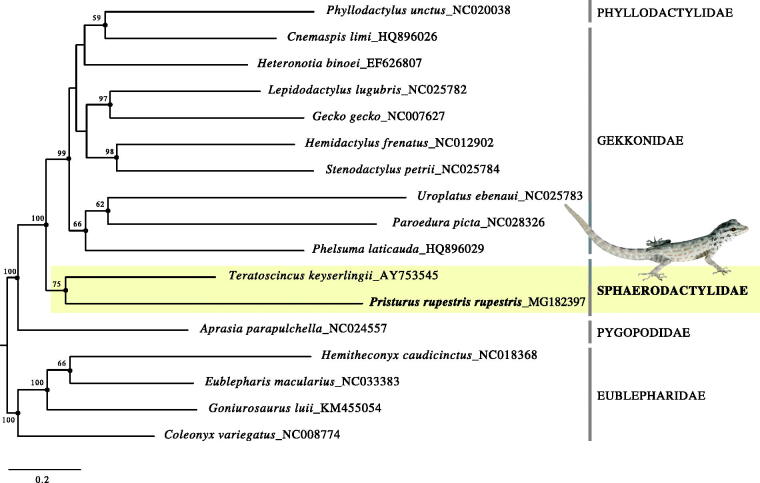
Phylogenetic relationships among *Pristurus r. rupestris* and representatives of 16 other Gekkota genera based on the concatenated sequences of 13 mitochondrial protein coding genes plus the two rRNAs (12S and 16S). Branch lengths and topology are from the Maximum Likelihood analysis. Nodal support next to the nodes is shown as bootstrap percentages from maximum likelihood and black dots indicate posterior probability values ≥0.95 from Bayesian Inference (see Supplementary material). The representatives of the family Eublepharidae were used as outgroups.
